# Motor, cognitive and mobility deficits in 1000 geriatric patients: protocol of a quantitative observational study before and after routine clinical geriatric treatment – the ComOn-study

**DOI:** 10.1186/s12877-020-1445-z

**Published:** 2020-02-06

**Authors:** Johanna Geritz, Sara Maetzold, Maren Steffen, Andrea Pilotto, Marta F. Corrà, Mariana Moscovich, Maria C. Rizzetti, Barbara Borroni, Alessandro Padovani, Annekathrin Alpes, Corinna Bang, Igor Barcellos, Ralf Baron, Thorsten Bartsch, Jos S. Becktepe, Daniela Berg, Lu M. Bergeest, Philipp Bergmann, Raquel Bouça-Machado, Michael Drey, Morad Elshehabi, Susan Farahmandi, Joaquim J. Ferreira, Andre Franke, Anja Friederich, Corinna Geisler, Philipp Hüllemann, Janne Gierthmühlen, Oliver Granert, Sebastian Heinzel, Maren K. Heller, Markus A. Hobert, Marc Hofmann, Björn Jemlich, Laura Kerkmann, Stephanie Knüpfer, Katharina Krause, Maximilian Kress, Sonja Krupp, Jennifer Kudelka, Gregor Kuhlenbäumer, Roland Kurth, Frank Leypoldt, Corina Maetzler, Luis F. Maia, Andreas Moewius, Patricia Neumann, Katharina Niemann, Christian T. Ortlieb, Steffen Paschen, Minh H. Pham, Thomas Puehler, Franziska Radloff, Christian Riedel, Marten Rogalski, Simone Sablowsky, Elena M. Schanz, Linda Schebesta, Andreas Schicketmüller, Simone Studt, Martina Thieves, Lars Tönges, Sebastian Ullrich, Peter P. Urban, Nuno Vila-Chã, Anna Wiegard, Elke Warmerdam, Tobias Warnecke, Michael Weiss, Julius Welzel, Clint Hansen, Walter Maetzler

**Affiliations:** 1 Department of Neurology, Christian-Albrechts-University of Kiel, Kiel, Germany; 2 Department of Clinical and Experimental Sciences, Neurology Unit, University of Brescia, Brescia, Italy; 3 Neurology Department, Centro Hospitalar do Porto, Porto, Portugal; 4 Movement Disorders Unit, Neurology Service, Internal Medicine Department, Hospital de Clínicas, Federal University of Paraná, Curitiba, Brazil; 5 Institute of Clinical Molecular Biology, Christian-Albrechts-University of Kiel, Kiel, Germany; 6 Department of Internal Medicine I, Christian-Albrechts-University of Kiel, Kiel, Germany; 7 Instituto de Medicina Molecular, Lisbon, Portugal. CNS-Campus Neurológico Sénior, Torres Vedras, Portugal. Laboratory of Clinical Pharmacology and Therapeutics, Faculty of Medicine, University of Lisbon, Lisbon, Portugal; 8 Medical Clinic and Policlinic IV, Ludwig-Maximilians-University of Munich, Munich, Germany; 9 Institute of Human nutrition, Christian-Albrechts-University of Kiel, Kiel, Germany; 10 Hasomed GmbH, Magdeburg, Germany; 11 Third Medical Clinic for Gastroenterology/Rheumatology, Städtisches Krankenhaus Kiel, Kiel, Germany; 12 Department of Urology, Christian-Albrechts-University of Kiel, Kiel, Germany; 13 Research Group Geriatrics Lübeck, Red Cross Hospital Geriatric Centre, Lübeck, Germany; 14 Department of Psychiatry and Psychotherapy, ZIP, Centre for Integrative Psychiatry, Kiel, Germany; 15 Department of Neurology, Ruhr-University Bochum, St. Josef-Hospital, Bochum, Germany; 16 Department of Neurology, Asklepios Klinik Barmbek, Hamburg, Germany; 17 Digital Signal Processing and System Theory, Faculty of Engineering, Christian-Albrechts-University of Kiel, Kiel, Germany; 18 Department of Cardiac and Vascular Surgery, Universitätsklinikum Schleswig-Holstein Campus Kiel, Kiel, Germany; 19 Department of Radiology and Neuroradiology, Christian-Albrechts-University of Kiel, Kiel, Germany; 20 Geriatric Clinic, Städtisches Krankenhaus Kiel, Kiel, Germany; 21 Department of Neurology, University Hospital Muenster, Muenster, Germany

**Keywords:** Balance, Body-worn sensors, Wearables, Comprehensive geriatric assessment, Executive function, Gait, Older adults, Quantitative assessment

## Abstract

**Background:**

Motor and cognitive deficits and consequently mobility problems are common in geriatric patients. The currently available methods for diagnosis and for the evaluation of treatment in this vulnerable cohort are limited. The aims of the ComOn (COgnitive and Motor interactions in the Older populatioN) study are (i) to define quantitative markers with clinical relevance for motor and cognitive deficits, (ii) to investigate the interaction between both motor and cognitive deficits and (iii) to assess health status as well as treatment outcome of 1000 geriatric inpatients in hospitals of Kiel (Germany), Brescia (Italy), Porto (Portugal), Curitiba (Brazil) and Bochum (Germany).

**Methods:**

This is a prospective, explorative observational multi-center study. In addition to the comprehensive geriatric assessment, quantitative measures of reduced mobility and motor and cognitive deficits are performed before and after a two week’s inpatient stay. Components of the assessment are mobile technology-based assessments of gait, balance and transfer performance, neuropsychological tests, frailty, sarcopenia, autonomic dysfunction and sensation, and questionnaires to assess behavioral deficits, activities of daily living, quality of life, fear of falling and dysphagia. Structural MRI and an unsupervised 24/7 home assessment of mobility are performed in a subgroup of participants. The study will also investigate the minimal clinically relevant change of the investigated parameters.

**Discussion:**

This study will help form a better understanding of symptoms and their complex interactions and treatment effects in a large geriatric cohort.

## Background

The demographic changes associated with increased life-expectancy have led to a substantial increase in older people suffering from multimorbidity with age-related neurological diseases and functional impairment [1–3]. A target-oriented and specific geriatric treatment designed by a multiprofessional and –disciplinary team including neurological expertise, addressing both the clinical relevant functional deficits and the individual needs of the patients, is urgently needed [4, 5]. Impaired gait, balance, cognitive functions and, consequently, reduced mobility and falls are among the most relevant age-related functional impairments associated with multimorbidity. At 70 years, the prevalence of gait disorders is about 35% and increases further with age [6]. About one third of people aged 65 years or above fall at least once a year [7]. Interestingly, the prevalence of falls among neurological patients is nearly twice as high as in the general population [8]. Of these patients, 5–10% develop serious injuries, e.g. fractures and head trauma [9, 10]. Delayed recovery from fall-related injury in geriatric patients often requires long-lasting inpatient stays with high resource costs [11–13] and the possibility of complications such as pneumonia. Moreover, long-term morbidity associated with fear of falling affect quality of life and mobility [14–16].

Cognition, particularly executive functions, are also often affected in older adults [17, 18] and can interfere with daily life activities and influence mortality rates. In an 8-year follow-up study [19], people with deficits in executive functions had a higher mortality rate than those without. One reason may be the reduced ability to manage multiple medical conditions [19]. Executive dysfunctions even affect intervention outcomes. For example, a recent study showed that baseline executive function performance predicted performance on the mobility tests after training in older adults [20].

A growing amount of epidemiological and pathophysiological studies suggests that motor and cognitive deficits interact and amplify each other [14, 18, 21, 22]. The interaction is not surprising as: (i) recent neuroimaging studies indicate a strong involvement of, e.g. the thalamus, basal ganglia, cerebellum, mesiotemporal areas and the frontal cortex in gait and balance performance [23, 24], and (ii) lesions in these areas are associated with falls, e.g. for Parkinson patients [25–27].

Physical activity may depend on brain integrity and influences geriatric conditions, such as frailty. A recent study indicates that physical activity interventions can reduce the prevalence and severity of frailty in elderly people [28]. A post-mortem study showed that white matter lesions of the brain explained 4% of the variance of physical frailty in 165 participants with a mean age at death of 88 years [29]. However, the interaction between physical activity and age-associated functional impairment, such as motor and cognitive deficits and frailty, remain largely unexplained and need further investigation. Research and clinical routine commonly use qualitative measures for the assessment of mobility, and motor and cognitive deficits, and these tools improved our understanding of these symptoms. However, these tools have numerous disadvantages, such as inaccuracy, high time expenditure and investigator dependency [30]. Due to the dynamic development in the fields of life sciences and technology, quantitative measures to evaluate impairment of gait, balance, cognitive functions and mobility –including mobile technology, so-called “wearables”- are increasingly available also for medical purposes. This technology can generate highly accurate outcome parameters for clinical studies and is even close to be implemented in the clinical routines [30–33].

The first mayor aim of this prospective, explorative observational multi-center study is therefore to explore quantitative markers of gait, balance and cognitive deficits in relation to routine clinical and specific geriatric parameters –as assessed with the comprehensive geriatric assessment (CGA)- in a large cohort of geriatric patients with predominantly chronic neurologic conditions. Detailed information beyond usual CGA parameters, e.g. gait variability, step characteristics, postural control and (semi-)quantitative cognitive parameters could substantially improve our understanding of geriatric conditions [30]. We will also determine the minimal detectable and clinical relevant change of many of the parameters investigated.

The second mayor aim of the study is to examine the association between executive and attentional deficits and the identified quantitative motor parameters in this vulnerable clinical cohort. We hypothesize that these cognitive deficits have predictive value for certain gait and balance deficits. The third mayor main aim is to evaluate the efficacy of an individualized geriatric inpatient treatment. The large dimension and multifaceted construction of the dataset will also allow many additional hypotheses to be tested.

Novel aspects of this study are (i) the recruitment of a prospective and large geriatric cohort, (ii) the coverage of a broad range of clinically relevant parameters, (iii) the identification of stabile quantitative parameters with clinical relevance, (iv) the evaluation of treatment response, (v) the definition of the minimal clinically relevant change (MCRC) of the investigated parameters, (vi) the inclusion of newest mobile technology for the assessment of mobility, motor functions and balance aspects using validated algorithms, and (vii) the assessment of this vulnerable cohort at places beyond the clinical environment.

## Methods/design

### Ethics

Ethical approvals have been obtained from the ethical committees of Kiel, Brescia, Porto, Curitiba and Bochum. The centres have submitted their proposals according to the principles of the Declaration of Helsinki. All participants will receive detailed oral and written information about the content and procedure of the study.

### Participants

The study will include geriatric patients aged 70 years and older, with and without neurological conditions [34–36]. Patients aged between 50 and 69 years will also be considered if they suffer from at least two chronic conditions [35]. Additional inclusion criteria are the ability to stand without personal aid for at least ten seconds and to walk at least three meters (walking aids permitted). Exclusion criteria are severe deficits in consciousness (clinical diagnosis), more than two falls during the previous week (fall risk during the assessment too high), five points or less in the Montreal Cognitive Assessment (MoCA) test [37, 38], history of or current drug abuse (except nicotine) and (corrected) visual acuity below 60% (assessed using a Sloan Letter Chart for three meter distance [39]). Magnetic Resonance Imaging (MRI) will be performed in a subset of patients having a clinical indication for this examination. Participants suffering from claustrophobia, or having pacemakers, defibrillators, targeted drug delivery systems, deep brain stimulation, vena cava filters, cochlear implants or any kind of ferromagnetic material within the body will not be considered. The cohort will include inpatients treated in University and General hospitals and geriatric rehabilitation centres.

### Procedure

**Fig. 1:**
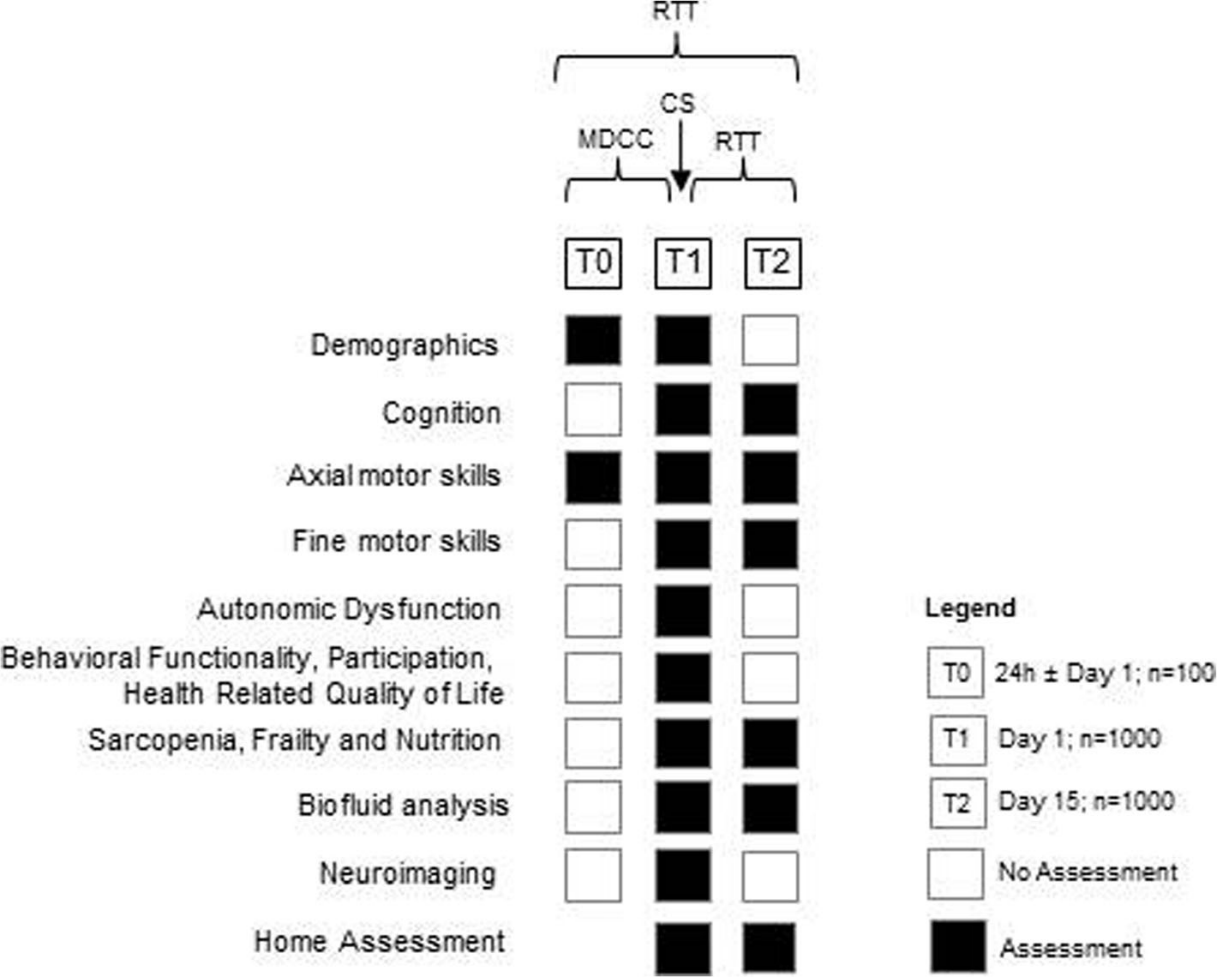
Study flowchart. Overview of the study including visits and relevant assessments. CS: Cross-sectional, MCRC: Minimal clinically relevant change, RTT: Response to treatment, T1: Baseline assessment (before / at admission), T2: Follow-up assessment (at / after discharge), T0: Time of assessment 0, for reliability / MCIC evaluation (24 h before or after T1)

This is a prospective, explorative observational multi-center study. Most of the participants will be recruited at admission. A subsample (*n* = 100) with a planned hospital stay (e.g. to evaluate new treatment options or to improve medication plans in severely affected patients that are at risk of losing functional independency) will be contacted via telephone, to ask them whether they would be interested to participate in a one week home-based assessment with wearables before and after the treatment phase. All participants will be assessed within the first two days (T1) and during the last two days before discharge (T2) of their inpatient stay. To determine the Minimal Detectable Change, an additional subgroup (*n* = 100) will undergo a visit (T0) 24 h before or after T1. Inpatient’s stay will be approximately 14–20 days. All participants will receive multidisciplinary care with an individually adapted set of therapeutic options depending on their needs during their inpatient treatment. Data obtained from T1 will be used to evaluate cross-sectional aspects of the study. Response to treatment will be evaluated by calculating the change between T1 and T2 after an approximately 14–20 days multidisciplinary treatment. Figure 1 illustrates the detailed study design.

### Measures

All participants will undergo an extensive and quantitatively oriented CGA, i.e. an assessment that collects information about all five relevant components of the International Classification of Functioning, Disability and Health (ICF) model [40]. Furthermore, a detailed evaluation of mobility, and specific motor and cognitive function will be conducted. For measurements of motor and cognitive parameters, translated and validated test versions will be used as far as available. Clinical and demographical data and questionnaires will also be assessed in the required languages.

#### Clinical and demographic data

Clinical and demographic data –including age, gender, diagnosis, initial and current symptoms, concomitant diseases, activities of daily living (ADL, [41]), instrumental ADL (iADL, [42]), nutritional aspects and medication– will be collected from clinical records and also with a semi-standardized clinical interview. Neurological routine assessment will include evaluation of strength (grip force), muscular proprioceptive reflexes, pallaesthesia, signs of ataxia, and frontal lobe dysfunction. We will use the *Geriatrie-Check,* which is a screening tool for the identification of geriatric patients [43, 44] and assesses aspects of dementia, level of care, frailty, and the premorbid level. It has recently been validated [44]. We will also use the geriatric screening according to Lachs et al. [45] to evaluate the functional aspects of vision, hearing, and urinary incontinence. Self-care and mobility skills (e.g. toilet use, eating, dressing, climbing stairs) will be appraised by the commonly used and reliable (kappa = 0.93) Barthel Index [46, 47]. Subjective improvement will be assessed using the Clinical Global Impression - Global Improvement - Scale (CGI-I, [48]).

Diagnoses and medication will be extracted from the medical reports. Extent of treatment and rehabilitation -as a covariate- will be evaluated using number and duration of therapeutic sessions as well as (change of) medication and medical aids.

#### Cognition

Cognitive functions will be measured with standardized neuropsychological screening tools and tests. We will use the MoCA for the evaluation of global cognitive performance. The MoCA has been shown to be internally consistent (Cronbach’s alpha = 0.83) and highly sensitive in detecting Mild Cognitive Impairment (MCI, 90%) and Alzheimer’s Disease (100%). Normative and validation data are available for Brazilian, Italian, German and Portuguese populations [38, 49–51]. For the assessment of frontal-executive dysfunctions the required version of the Frontal Assessment Battery (FAB) will be used [52–54]. The FAB consists of six items, testing aspects of conceptualization, lexical fluency, motor programming, sensitivity to interference, inhibitory control and environmental autonomy.

The Trail Making Test (TMT, [55]) assesses visual scanning and processing speed (TMT part A) as well as mental flexibility and divided attention ((TMT part B, B-A). Construct validity of the TMT is good [56] and there are normative data available stratified by age and education for the required languages [57–60].

In order to gain the second study aim in more detail regarding specific cognitive functions, the Kiel centre will perform a detailed neuropsychological testing in this subcohort, including the following tests:

- The *Testbatterie zur Aufmerksamkeitsprüfung* (*TAP,* [61]) is a computer-based assessment battery for attention. We will use the subtest “Alertness” to measure reaction time to a visual stimulus and the capability to inhibit reactions to a pre-stimulus.
- The standardized *Alters-Konzentrations-Test* (*AKT*, [62]) provides information about vigilance, concentration and focused attention (the capacity to focus on a stimulus while suppressing imposed distractors). Retest-reliability is high (r = 0.75–0.89, [63]).
- The Five-Point Test (FPT, [64]) is a standardized paper-pencil test for figural fluency and strategic thinking. The test consists of five-dot boxes in six rows on each sheet where participants produce as many different figures as possible by connecting the dots in each box within a defined time period. The FPT is a valid test that has excellent inter-rater (ICC = 0.99) and good test-retest reliability (ICC = 0.72–0.84, [64]).
- The *Regensburger Wortflüssigkeitstest (RWT,* [65]) assesses verbal fluency and flexibility. Subjects have to name as many words as possible within two minutes that (i) belong to a certain category, (ii) have a defined starting letter, (iii) belong to two different categories (alternating naming) and (iv) have two defined starting letters (again alternating naming). Inter-rater reliability of the test is excellent (ICC = 0.99) and test-retest reliability good (*r*_*tt*_ = 0.72 – *r*_*tt*_ = 0.89, [65]).
- The *Nürnberger-Alters-Inventar (NAI,* [66]), normed for people aged between 57 and 96 [66], provides information about cognitive and behavioural aspects. We will use the subtest *Farb-Wort-Interferenz-Test (FWIT)*, based on the widely used Stroop-Test, to assess attention and cognitive flexibility during provision of conflicting stimuli.

To avoid learning effects in T2, parallel versions of the MoCA, the AKT and RWT will be provided.

#### Axial motor function

**Fig. 2:**
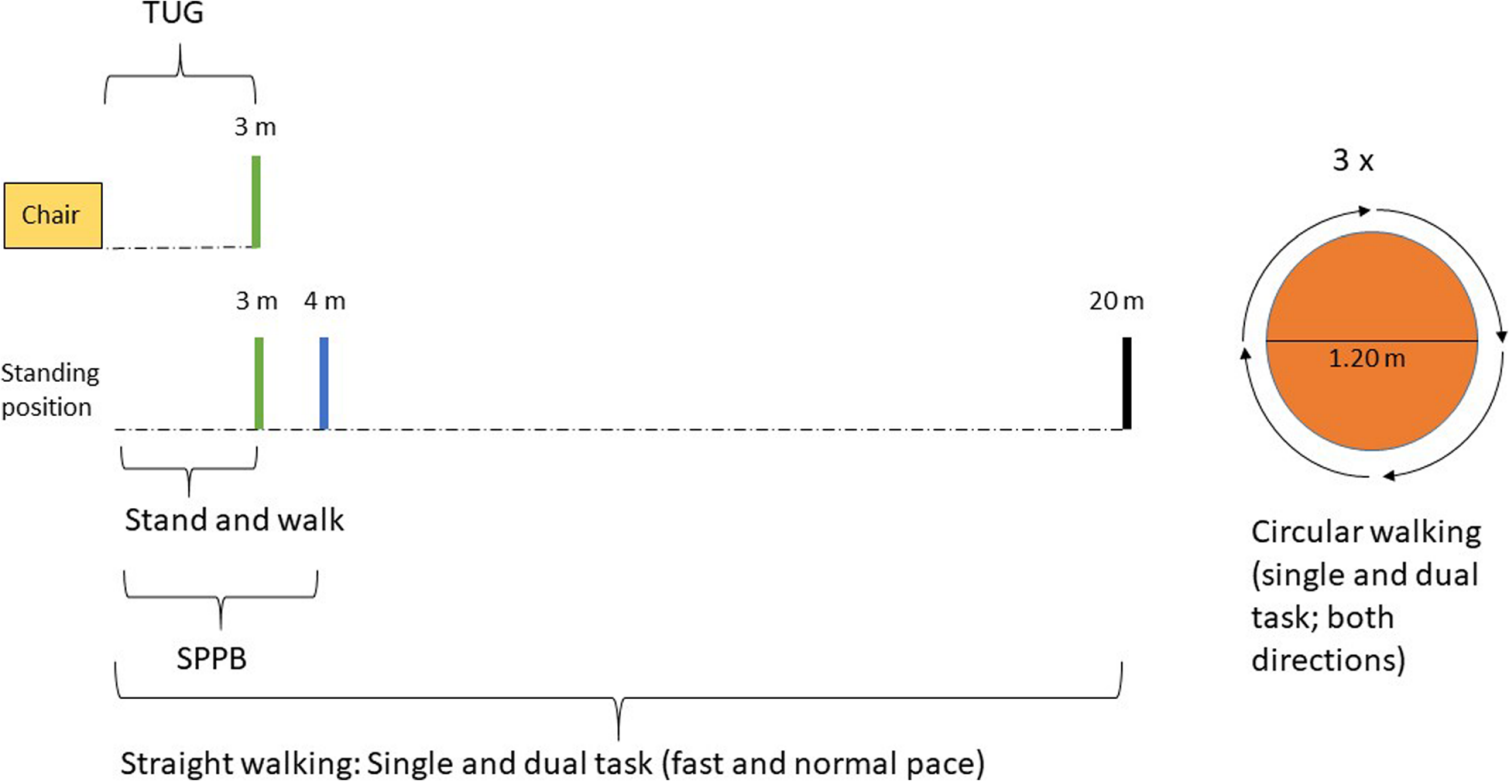
Assessment of axial motor function. Simplified illustration of the standardized motor tasks. SPPB: Short Physical Performance Battery, TUG: Timed-up-and-Go Test

Gait, balance and transfer aspects will be measured in a supervised environment (e.g., the ward, Fig. 2) using a set of well-established tests (summarized in Table 1), which will all be instrumented with CE-certified wearable devices (Rehagait®, Hasomed GmbH, Magdeburg, Germany; sensors at the feet and on the lower back).

**Table 1:** Tests of axial motor functions

Test	Task
Short Physical Performance Battery	Tandem, semi-tandem, side-by-side stand Two 4-m-walks with comfortable speed 5-Chair rise test, as fast as possible
Timed-up-and-Go test Straight walk Circular walk	Rise from a chair, 3-m-walk, turning, walk back Standing position, 3-m-walk, no turning Standing position, 20-m-walk under single and dual task conditions Walk around a 1.20 m circle under single and dual task conditions

The protocol will include the Short Physical Performance Battery (SPPB, [67–69]). The SPPB measures balance (tandem, semi-tandem, and side-by-side stand), gait speed (walking twice four meters at a comfortable speed) and chair rise performance (5-Chair rise test, as fast as possible) which has been shown to be reliable in older adults (ICC = 0.83–0.89, [70]). Participants will also perform the above-mentioned balance tasks on a foam pad (Airex balance pad, 50x41x6 cm). This test has already been performed under instrumented conditions with test-retest reliability (ICC) between 0.41 and 0.81 [71].

Moreover, the Timed-up-and-Go test (TUG) will be used to assess mobility aspects and turning. Recent studies suggest that instrumentation of the TUG with wearable devices can provide useful additional and complementary information to the generally used total time [68, 72–74].

Participants will also perform straight walks (out of a standing position) over three meters and 20 m and circular walks around a 1.20 m circle (360°). Single task performance will be assessed during both walking conditions with self-selected and as-fast-as-possible pace, except for circular walking (self-selected pace starting with the right leg and then with left leg). Dual task performance (checking boxes and subtracting serial 7 s) will be assessed during circular walks in self-selected pace condition, straight walking dual-task performance in fast pace condition [75–77].

The functional reach (FR) test measures balance at the limits of stability in the anterior direction. It can identify fall risk and balance impairment in geriatric patients [78, 79]. We have recently published an instrumented version of the test [79]. Participants will stand upright next to a wall with a yardstick and put their right arm in a stretched out position. Then they will reach forward as far as they are able to, and then be asked to keep this position for 15 s.

Part III of the revised version of the Unified Parkinson’s Disease Rating Scale (MDS UPDRS-III, [80]) will be used to assess axial deficits (e.g., via the postural instability and gait (PIGD) subscore) and parkinsonian signs. The Hoehn & Yahr scale will be used in patients with Parkinson’s disease (PD) to define disease severity [81].

The Falls Efficacy Scale (FES-I, [82]) consists of 16 questions about concerns regarding falling in specific activities of daily living (e.g., when getting dressed, when taking a shower or when shopping). The FES-I is a reliable instrument (Cronbach’s alpha = 0.79) and a strong association with previous as well as future falls has been found [82].

#### Fine motor function

The Functional Dexterity Test (FDT, pegboard test) is a reliable and valid instrument to measure finger and thumb movement [83]. Participants will turn 16 pegs in a zigzag manner as fast as possible on a wooden board with holes, first with the dominant, then with the non-dominant hand.

The 20-Cents test assesses fine motor skills under daily life conditions and is validated for geriatric patients [84]. Twenty 1-cent coins, spread over a white sheet of paper, will be picked up with each hand (first the dominant one, then the non-dominant one) and put into a box as fast as possible.

#### Health-related quality of life, behavior, social participation, physical activity and pain

Health-Related Quality of Life (HrQoL, [85, 86]) is one of the most important factors regarding treatment decisions and outcome of treatment. Main dimensions of HrQoL are physical, mental, social and role functioning. The EuroQol questionnaire with five dimensions (EQ-5D-5 L, [87]) consists of a descriptive part and the EQ Visual Analogue Scale. For the descriptive part, participants rate the impact of mobility and its deficits, self-care, usual activities, pain/ discomfort and anxiety/ depression on HrQoL. The EQ Visual Analogue Scale allows the participant to rate today’s overall HrQoL on a scale of 0 (worst health they can imagine) to 100 (best health).

The *Depression im Alter* scale (DIA-S, [88]) assesses specific aspects of depression and consists of ten items. The test person is asked to focus on the previous 14 days. The DIA-S is reliable (Cronbach’s alpha = 0.84) and has been validated in geriatric patients [89].

Apathy, a common symptom in neurological and psychiatric diseases, will be assessed with the German version of the Apathy Evaluation Scale (AES-D, [90]). The AES-D includes cognitive and emotional aspects of goal-directed behaviour. A total of 18 items are rated on a four-point Likert scale by the participant (AES-D-S, self-rated) and by a relative (AES-D-I, informant). The AES-D is reliable (Cronbach’s alpha = 0.91–0.94) and has been shown to be valid in patients with diverse diseases and in healthy adults [90].

The reliable (ICC = 0.70–0.94) and valid *Nürnberger-Alters-Alltagsaktivitäten-Skala (NAA)*, part of the *NAI* [66], is a 20-item questionnaire for the assessment of independency and participation in activities of daily living.

Physical activity (PA) will be assessed with the self-administered short version of the International Physical Activity Questionnaire (IPAQ, [91]). The participants are asked to estimate how much time in days per week and in hours per weekday they spend doing: (i) vigorous physical activities, (ii) moderate physical activities, (iii) walking, and (iv) sitting. Detailed information about reliability and validity are available for all versions from over twelve countries [91].

Pain will be assessed via the painDETECT Questionnaire (PD-Q), which is a reliable screening tool with high sensitivity, specificity and positive predictive accuracy [92].

#### Sarcopenia, frailty and malnutrition

The Jamar hydraulic hand dynamometer (AFH, Lügde, Germany) will be used to measure grip force [93]. Lean body / muscle mass and total body water and fat will be quantitatively assessed with the validated bioelectrical impedance analysis (BIA, Akern Bia 101, SMT medical GmbH & Co. KG, Würzburg, Germany, [94, 95]). The BIA will be applied as instructed in the manual with four electrodes (two at the right foot, two on the right hand) in a lying position after a rest phase of about ten minutes [96]. *For the definition of sarcopenia we will follow the definition of the European consensus on definition and diagnosis for sarcopenia* [97, 98].

Frailty will be assessed with the FRAIL-scale, a five-item questionnaire asking for fatigue, resistance, ambulation, illness and weight loss during the last three months. Usefulness for detecting frailty in elderly people has been proven [99–101].

The Swallowing Disturbance Questionnaire for Detecting Dysphagia (SDQ, [102]) is a 15-item questionnaire to detect dysphagia. The SDQ has been shown to be reliable (Cronbach’s alpha = 0.89) and useful to assess swallowing in PD.

Different aspects of malnutrition will be measured via interview using the Mini Nutritional Assessment (MNA, [103]), Malnutrition Universal Screening (MUST, [104]) and Subjective Global Assessment (SGA, [105]). The instruments assess nutritional status based on objective data (e.g. weight, height, Body-Mass-Index), physical examination and the participant’s self-report.

#### Autonomic dysfunction

At the location of Kiel, heart rate variability (HRV) will be examined using computer-assisted equipment (ProSciCard III, MediSyst GmbH, Germany) during rest and controlled deep breathing (six respiratory cycles per minute). Coefficient of variation, root mean square of successive differences, mean circular resultant, expiration-inspiration difference and E/I-ratio as well as a spectral analysis of HRV will be quantified and compared to age-related normal ranges of 120 healthy subjects [106].

Blood pressure (BP) and heart rate (HR) during orthostatic exposition will be monitored after ten minutes of supine rest on a tilt table. Patients will then be moved to the erect position (65°) and BP and HR changes recorded at one, three, and five minutes of head-up tilt. A decrease of systolic BP > 20 mmHg and diastolic BP > 10 mmHg within three minutes of tilting is regarded as orthostatic hypotension [107].

Residual urine volume will be determined with the BladderScan BVI6100 (Verathon Medical BV, The Netherlands, [108]). Moreover, the reliable and validated Qualiveen [109] questionnaire will be used for the evaluation of HrQoL in patients with urinary disorders. It covers frequency and intensity of different aspects (limitations, constraints, fears, feelings) of urinary dysfunction.

The German version of the Female Sexual Function Index (FSFI-d, [110]) is a 19-item questionnaire for the assessment of six different domains of female sexuality: desire, arousal, lubrication, orgasm, satisfaction, and pain. Its internal consistency (Cronbach’s alpha = 0.75–0.95) is good to very good. The International Index of Erectile Function (IIEF, [111]) is a self-administered questionnaire for males and includes aspects of erectile function, orgasm function, sexual desire, intercourse satisfaction and overall satisfaction. The original version (Cronbach’s alpha> 0.9, [111]) and the German translation have been shown to be reliable (Cronbach’s alpha = 0.95, [112]). As sexual function is a sensible topic also in older adults, participants will be informed again explicitly that answering this questionnaire is voluntarily.

#### Biofluid analysis

Participants will be asked to provide blood and stool samples for our established biobank [113]. Material will be collected from the wards and directly brought to the technicians responsible for the pre-processing and storage of the material, to ensure highest quality standards of the biosamples. Blood samples will be used for blood counts and DNA isolation, whereas stool samples will be used for gut microbiome analysis.

#### Neuroimaging

We will analyse gradient echo T1-weighted sequences, as well as T2-weighted flair sequences, susceptibility-weighted imaging and DTI datasets collected with a standardized protocol on a 3-T MRI. In addition, participants will be asked to provide any existing MRI data for semi-quantitative analysis [114].

#### Home assessment

Those patients, who will undergo a planned inpatient stay from a former clinical contact, will be contacted by phone in advance. Patients interested in joining the home assessment will be visited at their homes by staff and introduced into this part of the study. During the home assessment, participants will wear three wearables (inertial measurement units IMUs, GaitUp SA, Lausanne, Switzerland) fixed at the lower back and at the more affected ankle and wrist (if both sides are equally affected they wear the sensors on the right). They will also be asked to keep a structured diary about their activities to ensure comparability of subjective evaluation with IMU-based data. Participants will be assessed 24 h per day over seven days before and after the inpatient stay. In case patients may have difficulties with the handling of the sensor system, relatives will be asked to support the measurement process.

### Database and statistics

Study data will be collected and managed using REDCap electronic data capture tools hosted at Kiel University [115]. Statistical analysis will be performed using established statistical programs (e.g. R version 3.5.0, The R Foundation; SPSS 24, SPSS Corp, Chicago IL, USA). We abstained from providing a detailed analysis plan and power analyses as the analysis plans will be substantially influenced by the type of research question, and power analyses depend on both, concrete study hypotheses (which are given here only to a certain extent) and at least preliminary effect sizes that are, to our best knowledge, not yet available for most of the Parameters collected in this specific cohort. The use of z-scores will ensure comparability between data sets of different centres and countries. Common descriptive and inferential statistics and equivalent nonparametric statistics will be used for baseline data analysis. Logistic regression will be used to evaluate confounding factors (e.g. age, gender). A pre-post comparison with correction for multiple testing will be conducted to evaluate changes in mobility, motor function and cognition between T2 and T1. To assess reliability and responsiveness of the assessments (T1 to T0), we will use t-test and Cohen’s d after testing for normal distribution, and extract Intra-Class-Correlation (ICC), Standard Error of Measurement (SEM) and Minimal Detectable Change [116]. An explorative comparison of sensor-based data with clinical data and quantitative imaging parameters will be conducted by common descriptive and inferential statistics, (non-) parametric statistics and logistic regression.

## Discussion

This study will include 1000 geriatric patients, and this number may be increased in the course of the ongoing recruitment due to the exploratory, prospective, modular, and observational study design. We are not aware of a comparable endeavor in this research field. Due to the large number of participants, data obtained from this study will also allow sub-analyses focusing on, e.g., presence and absence of geriatric and non-geriatric conditions and comparisons across centers.

We will collect data covering many aspects of body structure and function, but will –in line with the CGA- go beyond this usually well-assessed ICF component and collect data of all five components of this WHO-designed and most widely accepted model of health and dysfunction [40]. The main asset of this study is in our view that as many as possible parameters of disability and symptomatology, from biofluid and neuroimaging, over quantitative geriatric syndrome assessment and parameters of autonomic and mobility dysfunctions will be collected on a quantitative level. The broad range of parameters will allow the use of novel analysis approaches and testing of hypotheses that can serve as an ideal starting point for the initiation of hypothesis-driven studies in the field of geriatrics.

This study will also evaluate treatment response through repeated assessment at the beginning and the end of multidisciplinary geriatric care programs. The programs will be comparable in the majority of participants and will encompass individual allied health training of at least 20 sessions, and re-evaluation and adaptation of medication (in the frame of, e.g., the *early rehabilitation in geriatric medicine* concept as applied in Germany [117, 118]). This approach will allow the definition of effective versus non-effective response-to-treatment parameters as well as the definition of predictive parameters for defined treatment approaches. This aspect is relevant especially at times when value-based healthcare [119, 120], precision medicine [121] and shared decision making [122] become increasingly important.

Moreover, we will evaluate test-retest reliability and minimal clinically relevant change through an additional T0 assessment. This approach is relevant in the light of the large number of assessments and inclusion of novel parameters in this study, to provide first evidence for the clinical meaningfulness of these parameters but also to provide information about the extent of noise that these parameters have during repeated assessments.

We will also use modern technology for the assessment of movement deficits, including but not limited to gait, balance, transfers, sleep and mobility. We will only apply algorithms that are validated for these populations for the extraction and appraisal of movement episodes and mobility patterns (e.g., [123–126]). It is expected that yet unknown symptoms will be detected that are not visible with the usual “clinical eye” [30]. We will evaluate our participants not only in the hospitals but will collect daily-life data during a 24/7 assessment before and after the inpatient stay in a subgroup. This approach will give us access to an entirely new field of research, i.e. mobility, movement, and behavioral aspects in the natural environment of the participants. These measures will provide complementary aspects to the supervised assessments in the clinic, where measures mainly reflect functional capacity (“How well can you perform?”), as parameters collected in the usual environment rather reflect functional activity (“How do you regularly perform?”) [127, 128]. We have recently learned that “identical” behaviors and movements can substantially differ depending on whether they are collected in the clinical or the home environment [129]. The home-based dataset will also allow evaluation of fluctuation in performance.

### Limitations

This study’s limitations include: firstly, the cohort includes old and frail people, and the assessment is somewhat exhaustive. Thus, it is possible that some participants lose motivation during the first assessment or between the first and second assessment. We will therefore split the respective assessments into parts and will allow adequate breaks (e.g. over lunch). This is possible as participants are investigated during an inpatient stay, and assessment times can be flexibly organized. Second, although the treatment is highly standardized at least in the German centers, this treatment is not comparable to standardized treatments as they are typically performed in clinical trials. Still, we feel that our approach is of value as this treatment reflects the “real life situation” in the participating centers and the high number of participants will most probably allow analyses in similarly treated subgroups. Third, the home assessment requires some technical understanding, which may not always be given in all participants. We will address this issue by asking spouses and other related people to help with the charging of the sensors, and by providing telephone contact in case technical issues occur. Fourth, use of novel technology always includes the risk of technical problems and potential data loss. We are confident that this is a little risk as we have long-lasting experience with the companies providing the sensors and constant communication and support is ensured by the manufacturers. Finally, our multi-center design requires an intense and regular interaction between respective principle investigators and study personnel, and highly standardized protocols. We address these aspects by providing all relevant documents in English, by performing personal visits at all cites to personally train the assessments and to solve any upcoming issues, and by regularly and randomly performed internal quality checks of the data.

This exploratory study investigates a large sample of geriatric patients. It uses a comprehensive, mainly quantitative and novel technology-oriented assessment protocol that is performed in the clinic and at home and thus goes beyond the already established CGA. This study design will allow evaluation of treatment effects. Taken together, this study has the potential to enhance our understanding of geriatric deficits and the intra-individual interaction of neurological age-related diseases. The dataset will also allow drawing new conclusions and hypotheses about disease and treatment effects in this vulnerable population.

## Data Availability

Not applicable.

## Abbreviations

ADL: Activities of daily living
AES-D: Apathy Evaluation Scale - German Version
AKT: Alters-Konzentrations-Test
BIA: Bioelectrical impedance analysis
ComOn: Cognitive and Motor Interaction in the Older Population
DIA-S: Depression im Alter - Skala
EQ-5D-5 L: EuroQoL questionnaire (5 dimensions, 5 level of answers)
FAB: Frontal Assessment Battery
FDT: Functional Dexterity Test
FES-I: Falls Efficacy Scale - International
FPT: Five-Point Test
FR: Functional reach
FSFI-d: Female Sexual Funtion Index – German version
FWIT: Farb-Wort-Interferenz-Test
HrQoL: Health-Related Quality of Life
iADL: Instrumental activities of daily living
ICC: Intra-class-correlation-coefficient
IIEF: International Index of Erectile Function
IPAQ: International Physical Activity Questionnaire
MARDS: Montgomery-Asberg Depression Scale
MCI: Mild Cognitive Impairment
MDS UPDRS: Revised version of the Unified Parkinson’s Disease Rating Scale
MNA: Mini Nutritional Assessment
MoCA: Montreal Cognitive Assessment
MRI: Magnetic resonance imaging
MUST: Malnutrition Universal Screening Tool
NAA: Nürnberger-Alters-Alltagsaktivitäten-Skala
NAI: Nürnberger-Alters-Inventar
PA: Physical activity
PD: Parkinson’s Disease
PD-Q: paindetect questionnaire
PIGD: Postural instability and gait
RWT: Regensburger Wortflüssigkeitstest
SDQ: Swallowing Disturbance Questionnaire
SEM: Standard error of measurement
SGA: Subjective Global Assessment
SPPB: Short Physical Performance Battery
T0: Time of assessment 0
T1: Time of assessment 1
T2: Time of assessment 2
TAP: Testbatterie zur Aufmerksamkeitsprüfung
TMT: Trail Making Test
TUG: Timed-up-and-Go test
